# Continuous thrombocytopenia after SARS-CoV-2 nucleic acid negative in a non-severe COVID-19 patient for several months

**DOI:** 10.1186/s12879-020-05495-5

**Published:** 2020-10-19

**Authors:** Xia Wu, Dongxia Luo, Yaling Liu, Yilan Zeng, Yuping Gong

**Affiliations:** 1 Department of Hematology, West China Hospital, Sichuan University, Chengdu, Sichuan Province China; 2 Department of Internal Medicine, The Public and Health Clinic Centre of Chengdu, Chengdu, China

**Keywords:** Non-severe COVID-19, Continuous thrombocytopenia, SARS-CoV-2

## Abstract

**Background:**

Thrombocytopenia was common in the coronavirus disease (Covid-19) patients during the infection, especially in severe COVID-19 patients, but was less in the non-severe Covid-19 patients. However, the platelet count would be restored after antivirus treatment. In this paper, we report continuous thrombocytopenia in a non-severe Covid-19 case after a negative nucleic acid test for Covid-19.

**Case presentation:**

A non-severe COVID-19 patient had the platelet continuous decrease for several months after the SARS-CoV-2 nucleic acid turning negative, and without well response to the glucocorticoid. The dynamic change of platelet count followed that of the lymphocyte count. After excluding the medicines possibility, immune system disorders, other specific virus infection and specific antibody of platelet, the thrombocytopenia continuously lasted for several months. The upward trend did not begin until June 2020 and she took the tapering dose of prednisone under the instruction of the hematologist.

**Conclusion:**

Excluding other potentialities inducing thrombocytopenia, we highly hypothesized the SARS-CoV-2 may cause thrombocytopenia by disturbing the immune system to induce the thrombocytopenia in our report,, which needs longer time to restore the immune system and platelet count via the glucocorticoid. We firstly reported this case in order to contribute the clinician to better deal with the patients like this.

## Background

In December 2019, a cluster of unknown acute respiratory illness occurred in Wuhan, the capital city of Hubei province in China. Now the disease known as coronavirus disease (COVID-19) caused by severe acute respiratory syndrome coronavirus 2 (SARS-CoV-2) has since been declared a pandemic by the Word Health Organization on March 11, 2020 [1]. Fever, dry cough, fatigue, anorexia and dyspnea are common symptoms in patients with COVID-19, and the human-to-human transmission of COVID-19 has been confirmed [2]. Like SARS-CoV and MERS-CoV, SARS-CoV-2 has also been reported to induce thrombocytopenia, especially in severe COVID-19 [3–8], but the platelet count is restored after timely antivirus treatment. However, there is no report about significant reduction of platelets in non-severe COVID-19 patients following SARS-CoV-2 nucleic acid turning negative. Here, we report our first non-severe Covid-19 case of continuous thrombocytopenia after a negative nucleic acid test for Covid-19. We hypothesized that the continuous thrombocytopenia in this case was due to immune dysregulation, especially the T cells, such as CD4+ T cells and CD8+ T cells, which was important for the immune system. As reported in the previous studies, T cells play a vital role in immune system and may affect the effective of glucocorticoid on the thrombocytopenia [9]. We defined the degree of severity of Covid-19 at the time admission according to the seventh Trial Version of the Novel Coronavirus Pneumonia Diagnosis and Treatment Guidance. The definition of continuous thrombocytopenia was the platelet count below 150 or 100◊10^9^/L for three to twelve months.

## Case presentation

A 49-year-old woman had a history of fever on 2nd February 2020 after coming in contact with her husband who had been diagnosed with Covid-19. At that time, two throat and nasal swabs of SARS-CoV-2 nucleic acid were negative and the chest CT was normal, and the platelet count was 103◊10^9^/L. On 9th February 2020, the woman was nucleic acid positive for Covid-19 after a bronchoalveolar lavage, and diagnosed with non-severe Covid-19 according to the seventh Trial Version of the Novel Coronavirus Pneumonia Diagnosis and Treatment Guidance. She was admitted to the designated hospital (the Public and Health Clinic Centre of Chengdu) to receive treatment. In the designated hospital, all reexamination indicators were normal except the absolute lymphocyte count and C-reactive protein (CRP) (Table 1). The initial treatment mainly included lopinavir/ritonavir tablets (500 mg bid). After an eight-day therapy in hospital, the nucleic acid test for SARS-CoV-2 turned negative for two consecutive tests, and other laboratory tests were normal as well (Table 1). Therefore, she was discharged from hospital on 18th February 2020.

**Table 1:** The follow-up observation indicators of the patient

Parameters	First admission	Discharge	First review	Second review	Third review	Forth review
	10 Feb	18 Feb	4 Mar	19 Mar	20 Apr	18 May
white blood count(3.5–9.5) x10^9^/L	4.7	6.23	5.76	6.34	8.86	10.71
neutrophils (2–7) x10^9^/L	3.26	3.67	3.35	3.89	5.44	6.47
lymphocyte (0.8–4) x10^9^/L	1.12	1.98	1.93	1.94	2.84	3.78
hemoglobin (110-160 g/L)	117	114	133	146	133	143
platelet count (100–300) x10^9^/L	121	192	45	15	62	68
Albumin (35-55 g/L)	36.6	35.6	45.3	53.5	41.6	42.1
globulin (20-35 g/L)	26.1	24.1	31.4	35.7	24.2	20.8
Alanine aminotransferase (> 37 U/L)	16	17	30	48	19	22
Aspartate aminotransferase (> 37 U/L)	20	20	25	42	17	20
Total bilirubin(> 20.5umol/L)	2.8	4.4	10.3	12.1	9.6	8.2
Creatinine (40-133umol/L)	46	59	53	53	62	61
Lactate dehydrogenase (109-245 U/L)	214	158	159	203	156	216
creatine kinase (25-196 U/L)	44	59	41	46	20	32
D-dimer (0-1μg/ml)	1.22	NA	0.66	0.56	0.43	0.49
Fibrinogen (0-5μg/ml)	4.1	NA	2.2	1.7	1.6	NA
C-reactive protein (0-5 mg/L)	86.34	1.3	0.83	1.29	< 0.8	0.83
SAA (0-10 mg/L)	> 320	18.08	< 4.8	< 4.8	< 4.8	4.93
Procalcitonin> = 0.5 ng/ml	0.051	NA	NA	NA	NA	NA
**Lymphocyte subsets (cells/ul)**						
CD3 + T cells (770–2041)	736	1459	1285	1018	1654	2897
CD4 + T cells (414–1123)	490	905	734	588	1163	1710
CD8 + T cells (238–874)	212	434	412	346	415	936
CD4/CD8	2.31	2.09	1.78	1.7	2.8	1.83
CD19 + B cells (90–560)	129	125	166	126	372	370
CD56 + NK cells (150–1100)	45	227	208	249	150	302
**nucleic acid of SARS-CoV-2 (+/−)**	+	–	–	–	–	–
**Change of the chest CT**	**Ab**	**S**	S	S	S	N
**Novel coronavirus specific antibody**						
IgG (+/−)	NA	NA	NA	NA	+	+
IgM (+/−)	NA	NA	NA	NA	–	–

The follow up of laboratory indicators were in this table from the first admission, mainly including the peripheral blood count, biochemistry indicators, lymphocytes and the result of nucleic acid for SARS-CoV-2. the follow-up was carried out every 15 days after the first discharge.

*+* positive, *-* negative, *NA* no available, *SAA* serum amyloid A, *Ab* abnormal, *S* stable or absorption

Fourteen days after discharge, the patient presented with petechiae on the limbs, without mucous membrane bleeding (epistaxis or gum bleeding). But she did not go to see a doctor. Until on 4th March 2020, she accepted the regular review of laboratory tests and chest CT in the designated hospital, indicating that the platelet count was 45◊10^9^/L but other blood parameters were all normal (Table 1). The autoimmune antibody test was negative, including the anti-nuclear antibody (ANA), anti-Sm antibody, anti-U1-nRNP antibody, anti-SSA antibody, anti-SSB antibody, and other myositis antibodies. The hepatitis B and C virus markers were negative. Therefore, the patient was instructed by the doctor in the designated hospital to start to take Leucogen (20 mg tid) in the outpatient clinic for 2 weeks to increase platelet. This medicine, a cysteine derivative, could enhance the function of the bone marrow hematopoietic system to increase the platelet count. However, the purpura occurred on the lower limbs again after 7 days, she did not go to see a doctor until on 19th March 2020 for the second follow-up review. The platelet count was 15◊10^9^/L, the purpura was also limited to the lower limbs at the same time, the CD4+ T cells reduced to 588 from 780 and CD8+ T cells from 412 to 346, but the other laboratory results were normal (Table 1). Given the severe thrombocytopenia, the patient was readmitted immediately in the designated hospital and hematologist prescribed 10 mg dexamethasone intravenously and platelet transfusion to improve platelet to at least 30◊10^9^/L. After the above treatment, the platelet count raised to 56◊10^9^/L next day, then prednisone 25 mg twice a day with a gradually reduced dose to maintain the number of platelet about at 50◊10^9^/L. Due to the recovery of platelet count, she was arranged to discharge on 20th March 2020.

**Fig. 1:**
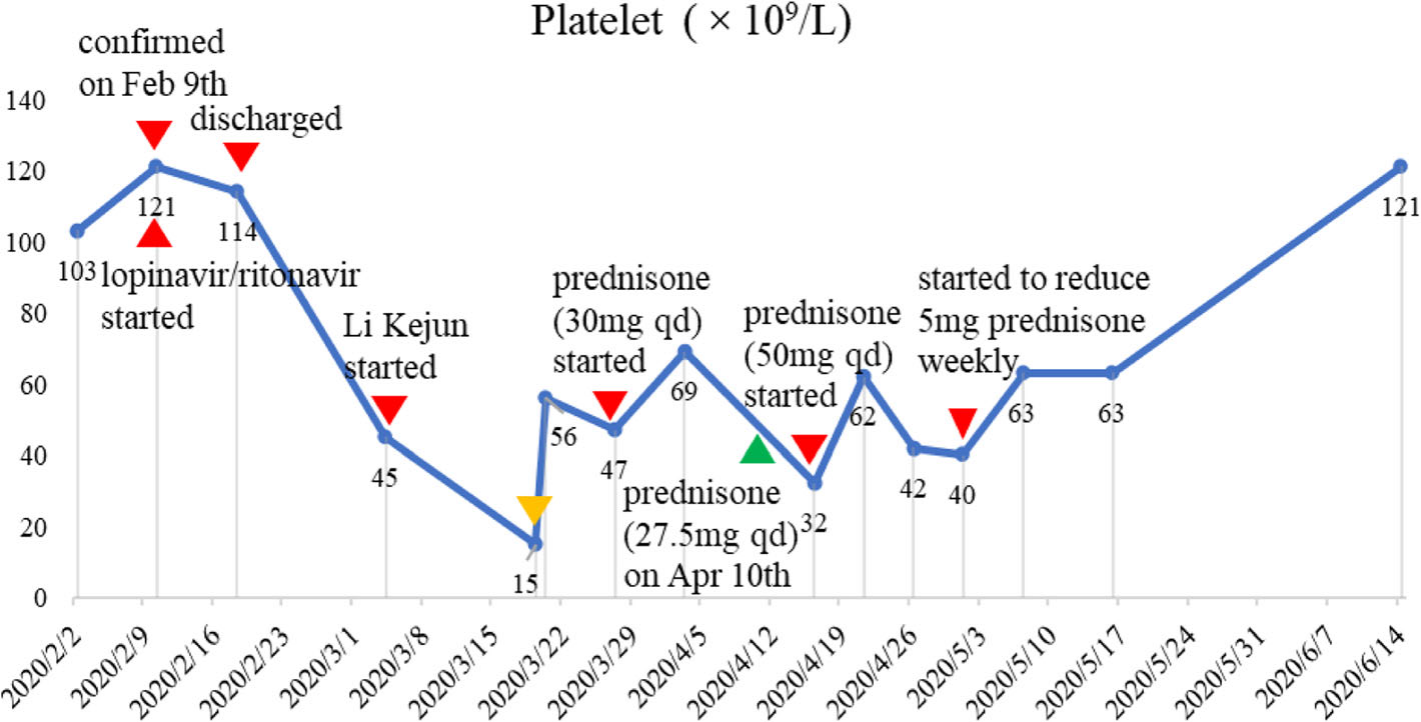
The change of platelet count and dose of prednisone. The orange triangle on the line means the time when 10 mg of dexamethasone IV were given and platelets transfused. The green triangle on the line means on 10 April 2020, the dose of prednisone was tapered down from 30 mg to 27.5 mg, but without the platelet examination

On 27th March 2020, she went to see a hematologist with platelet count being 47 ◊10^9^/Land started to increase the dose of prednisone to 30 mg/d till 10th April 2020 under the instruction of the hematologist. The platelet count increased to 69◊10^9^/L on 3rd April 2020, therefore, the dose of prednisone was tapered down from 30 mg to 27.5 mg on 10th April 2020. But on 16th April 2020, she complained of fatigue and general malaise and the platelet count was found to be 32◊10^9^/L, therefore, the dose of prednisone increased to 50 mg per day.. On April 21th, April 26th and May 1st, the platelet count was 62◊10^9^/L, 42◊10^9^/L and 40◊10^9^/L, respectively. Under the instruction of the hematologist, she started to reduce 5 mg of prednisone every week until 28 May 2020, and then reduce the 2.5 mg dose every week. Due to the continuous reduction and instability of platelet count, hematologist advised her to complete a palate specific antibody test. She accepted the special antibody of platelet with all negative results, including GPIb/IX, GPIV, HPA-1a, 3a, 4a and HPA-1b,3b, 4a, HPA-5b,5a. The latest test of platelet was 121 g/L on forth June 2020, and now she takes 17.5 mg prednisone per day under the instruction of the hematologist (Fig. 1).

This patient, an employee in an education company, had no history of allergies, hepatitis and surgery. And she had no immune diseases, hyperthyroidism, chronic gastritis, diabetes, hypertension, heart disease, chronic lung disease, etc. The human immunodeficiency virus (HIV), hepatitis B and C virus, syphilis and venereal disease research laboratory tests were all negative (Table 2).

**Table 2:** Basic characteristics and personal history

Characteristics		Medicine history	
Age	49	NSAIDS	
Sex	female	antibiotics	N
Family history	N	Immunosuppressors	N
**Medical history**		**Specific tests**	N
immune diseases	N	Hepatitis B virus	
chronic gastritis	N	Hepatitis C virus	N
hyperthyroidism	N	HIV	N
diabetes	N	Syphilis	N
hypertension	N		N
COPD	N		
chronic liver disease	N		
chronic renal disease	N		

We exclude the potential factors inducing the thrombocytopenia in our report as shown in this table

*N* no or negative for test, *COPD* chronic obstructive pulmonary disease, *NSAIDS* Non-Steroidal Anti-inflammatory Drugs

## Discussion and conclusion

Many factors can induce secondary thrombocytopenia, such as immune disease, disseminated intravascular coagulation (DIC), thrombotic thrombocytopenic purpura (TTP), and antibiotics [10]. Among them, Virus-infection is a common cause. Like SARS-CoV and MERS-CoV viruses [3], SARS-CoV-2 may induce thrombocytopenia and the mechanism includes abnormal immune function, directly impairing bone marrow hemopoiesis [11, 12] and lung damage [13]. Previous studies have reported thrombocytopenia in patients with COVID-19 during the infection [14–16], but most of them will gradually recover after antivirus treatment and also the thrombocytopenia was more common in patients in ICU or non-survivors [5, 6]. However, in our report, a non-severe COVID-19 patient without comorbidities and significant lung injury had significant and continuous thrombocytopenia after the nucleic acid for SARS-CoV-2 was negative. The three reexaminations of nucleic acid for SARS-CoV-2 were all negative and the last examination of the specific antibody for SARS-CoV-2 indicated that IgM was negative and IgG was positive, indicating the patient suffered from SARS-CoV-2. After taking prednisone for a long time, the platelet count remained low until 4th June 2020, therefore, we highly suspected that the patient had a secondary ITP caused by SARS-CoV-2 infection. Lymphocytes and subsets play a vital role in the maintenance of immune system [9]. The decrease of the lymphocyte and subsets, especially the CD4+ T cells, CD8+ T cells and NK (CD56+) cells was significant, indicating an impairment of the immune system caused by SARS-CoV-2 infection, which might trigger an autoimmune response [17]. CD4+ T cells help B cells to produce virus specific antibodies and CD8+ T cells can kill the virus-infected cells by cytotoxicity. Therefore, the depletion and dysfunction of the lymphocytes and their subsets induced the immune system abnormal resulting of thrombocytopenia. We also found that the dynamic characteristic of the platelet count was in consistent with that of the lymphocyte and subsets. Therefore, we deduced that the SARS-CoV-2 might disturb the immune system to induce the thrombocytopenia. Even if nucleic acid of SARS-CoV-2 negative, damage of the immune system might still be present to induce the continuous thrombocytopenia. Now the platelet count has gradually recovered after taking prednisone for about 3 months.

In our paper, excluding other potential factors, we supposed the patient was secondary ITP caused by SARS-CoV-2 infection impairing the immune system. This damage needs more time to recovery the immune system and platelet count via the glucocorticoid treatment. Although, thrombocytopenia was common in patients with COVID-19, the continuous decrease of platelet in patients after SARS-CoV-2 nucleic acid turning negative was not, which may contribute the clinicians to deal better with these patients. What’s more, this rare phenomenon might lead physicians to explore the potential mechanism causing the platelet decrease.

## Data Availability

All data generated or analyzed during this study are included in this published article.

## Abbreviations

COVID-19: Coronavirus disease
SARS-CoV-2: Severe acute respiratory syndrome coronavirus 2
ITP: Immune thrombocytopenia
CRP: C-reactive protein
HIV: Human immunodeficiency virus
SAA: Serum amyloid A
ANCA: Antinuclear antibody
DIC: Disseminated intravascular coagulation
TTP: Thrombotic thrombocytopenic purpura
